# Zearalenone lactonohydrolase activity in *Hypocreales* and its evolutionary relationships within the epoxide hydrolase subset of a/b-hydrolases

**DOI:** 10.1186/1471-2180-14-82

**Published:** 2014-04-03

**Authors:** Delfina Popiel, Grzegorz Koczyk, Adam Dawidziuk, Karolina Gromadzka, Lidia Blaszczyk, Jerzy Chelkowski

**Affiliations:** 1 Institute of Plant Genetics Polish Academy of Sciences, Poznan, Poland; 2 Department of Chemistry, Poznań University of Life Sciences, Poznan, Poland

**Keywords:** Zearalenone lactonohydrolase, Zearalenone, *Clonostachys* sp, *Trichoderma* sp, *Fusarium* sp, Mycotoxins, Epoxide hydrolase, Homology modelling

## Abstract

**Background:**

Zearalenone is a mycotoxin produced by several species of *Fusarium* genus, most notably *Fusarium graminearum* and *Fusarium culmorum*. This resorcylic acid lactone is one of the most important toxins causing serious animal and human diseases. For over two decades it has been known that the mycoparasitic fungus *Clonostachys rosea* (synonym: *Gliocladium roseum*, teleomorph: *Bionectria ochroleuca*) can detoxify zearalenone, however no such attributes have been described within the *Trichoderma* genus.

**Results:**

We screened for the presence of zearalenone lactonohydrolase homologs in isolates of *Clonostachys* and *Trichoderma* genera. We report first finding of expressed zearalenone lactonohydrolase in *Trichoderma aggressivum*. For three isolates (*T. aggressivum, C. rosea* and *Clonostachys catenulatum* isolates), we were able to reconstruct full coding sequence and verify the biotransformation ability potential. Additionally, we assessed progression of the detoxification process (in terms of transcript accumulation and mycotoxin decomposition *in vitro*).

*In silico,* search for origins of zearalenone lactonohydrolase activity in model fungal and bacterial genomes has shown that zearalenone lactonohydrolase homologs form a monophyletic fungal clade among the a/b hydrolase superfamily representatives. We corroborated the finding of functional enzyme homologs by investigating the functional sites (active site pocket with postulated, noncanonical Ser-Glu-His catalytic triad) conserved in both multiple sequence alignment and in homology-based structural models.

**Conclusions:**

Our research shows the first finding of a functional zearalenone lactonohydrolase in mycoparasitic *Trichoderma aggressivum* (an activity earlier characterised in the *Clonostachys rosea* strains). The supporting evidence for presence and activity of functional enzyme homologs is based on the chemical analyses, gene expression patterns, homology models showing conservation of key structural features and marked reduction of zearalenone content in cultured samples (containing both medium and mycelium). Our findings also show divergent strategies of zearalenone biotransformation ability (rapid induced expression and detoxification vs. gradual detoxification) present in several members of *Hypocreales* order (*Trichoderma* and *Clonostachys* genera). The potential for lactonhydrolase activity directed towards zearalenone and/or similar compounds is likely ancient, with homologs present in several divergent filamentous fungi among both *Sordariomycetes* (*Bionectria* sp., *Trichoderma* sp., *Apiospora montagnei*) and *Leotiomycetes* (*Marssonina brunnea* f. sp. ‘multigermtubi’).

## Background

The resorcylic acid lactones are mainly produced by fungi belonging to *Hypocreales* order (e.g. *F. graminearum*, *Hypomyces subiculosus*, *Pochonia chlamydosporia*). Majority of the known compounds is bioactive [1]. The most widespread (due to its potential for accumulation in food and feed) is zearalenone (6-(10-hydroxy-6-oxo-trans-1-undecenyil)-resorcylic acid lactone).

Zearalenone (ZEN) - a mycotoxin produced by several species of *Fusarium,* most notably *F. graminearum* and *F. culmorum -* has relatively low acute toxicity, but it exhibits distinct estrogenic and anabolic properties [2], due to its ability to couple with the estrogen receptor. The examination of ZEN metabolism in swine, rat and chickens liver resulted in α- and β-zearalenol as products (the latter compound being recognized as non toxic) with indication that α-zearalenol binds to estrogen receptors 10–20 times stronger than ZEN and 100 times stronger than β-zearalenol. The long-term effects of ZEN exposure include genotoxic and carcinogenic effects e.g. [3,4], as well as variety of reproductive disorders in animals e.g. [5-7].

*In vivo*, zearalenone has been proven to exhibit significant fungistatic effects and is thought to contribute one of the key mechanisms of competition between producer and non-producer species [8]. In keeping with this, ability to detoxify zearalenone is thought to confer a considerable adaptive advantage to competing fungal taxa [9].

Among the fungi of *Hypocreales* order, the mycoparasitic fungus *C. rosea* was long known to degrade zearalenone [10]. The exact mechanism of detoxification was determined in form of zearalenone-specific lactonase (zearalenone lactonohydrolase) enzyme (*zhd101*) which catalyzes the hydrolysis of ZEN, a process followed by spontaneous decarboxylation [11]. The end products exhibit both significantly lessened toxic effects and a decreased affinity for estrogen receptors.

To this day, independent detoxification mechanisms have been reported both in fungi (*Trichosporon mycotoxinivorans*) [12] and in bacteria (*Rhodococcus pyridinivorans*) [13]. However, a systematic screening of potential biocontrol agents (divergent fungi of *Hypocreales* order - mainly *Clonostachys* sp. and *Trichoderma* sp.) for lactonohydrolase activity and expression patterns has not, to our knowledge, been described in literature.

In this study, we present the results of screening a combined collection of *Trichoderma* and *Clonostachys* isolates, for strains with functional lactonohydrolase homologs and confirmed biotransformation ability. We report the first finding of a functional zearalenone lactonohydrolase in *T. aggressivum*. We also present results of an inquiry into the evolutionary basis of potential resorcyclic acid lactonohydrolase activity in filamentous fungi.

## Results

### Population screening for potential biocontrol agents

Taxonomic identification of isolates used in the screening was carried out with use of both morphological (mycelium and conidia morphology) and molecular techniques (ITS and TEF sequences; Th2/Th4 marker [14]).

**Figure 1:**
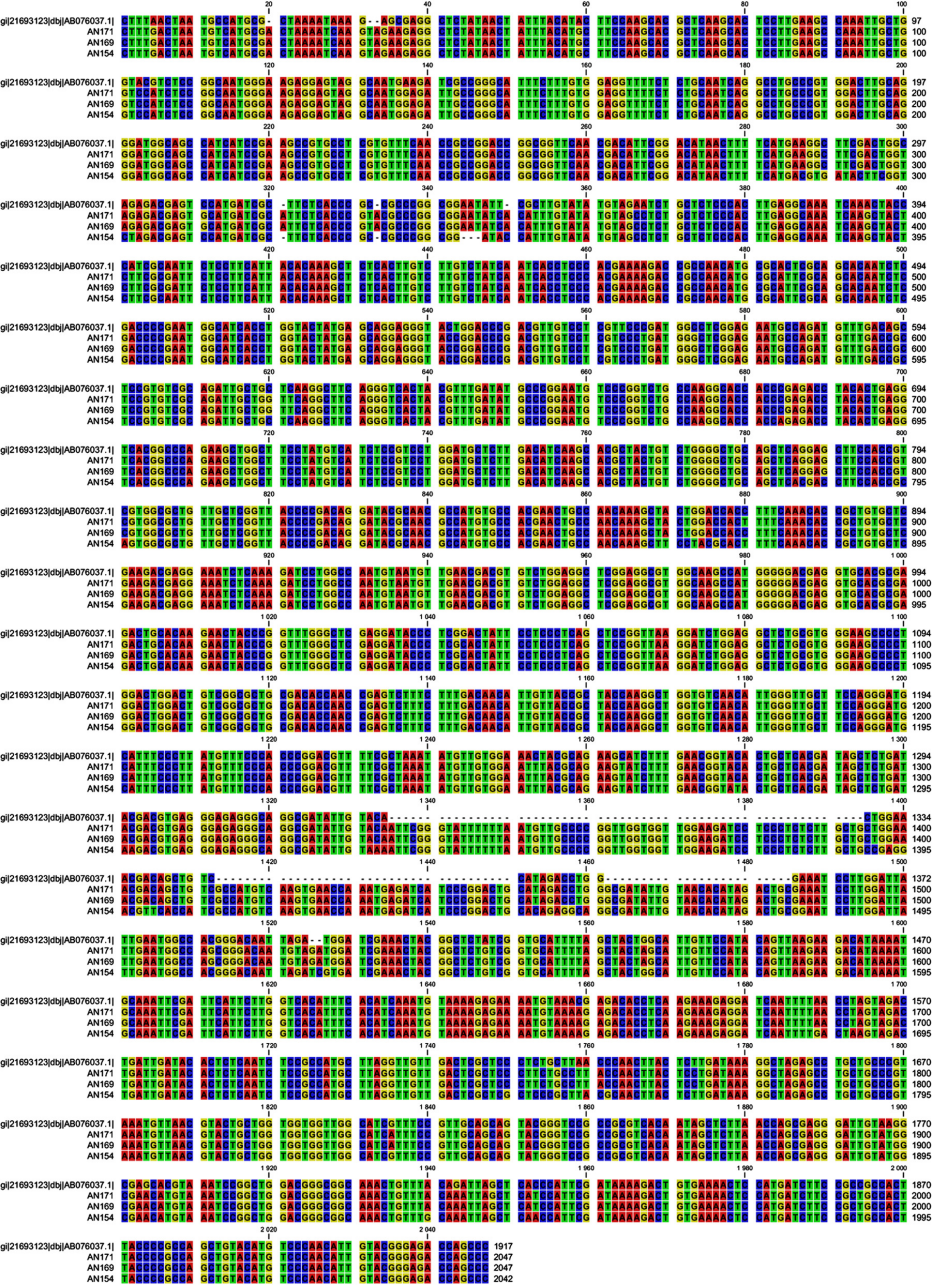
**Comparative analysis of the zearalenone lactonohydrolase gene sequence in the *Trichoderma *and *Clonostachys *isolates compared to the complete sequence of the model gene *C. rosea *AB076037.** AN 171, AN 169, AN 154 isolates with identified sequences homologous to the zearalenone lactonohydrolase gene, origin - the sequence of the model gene - AB076037.

We found seven pairs of primers amplifying overlapping products nested within the zearalenone lactonohydrolase coding sequence (products of ca. 300 bp). Total of seventy nine isolates belonging to the *Trichoderma* and *Clonostachys* genera were tested for the presence of the gene. For three isolates (*C. catenulatum* - AN 169, *C. rosea -* AN 154 and *T. aggressivum -* AN 171) we obtained full length products for all seven primer pairs, which allowed for the assembly of complete coding sequences for respective lactonohydrolase homologues (see Figure 1 for multiple alignments with model *Clonostachys* sequence as well as reference model species sequences). The three isolates were further investigated in detail. GenBank accession numbers: AN 169 - KF 515222, AN 154 - KF 515223, AN 171 - KF 515221.

### Verification of biotransformation ability potential in isolates of Clonostachys sp. and isolate of Trichoderma sp

**Figure 2:**
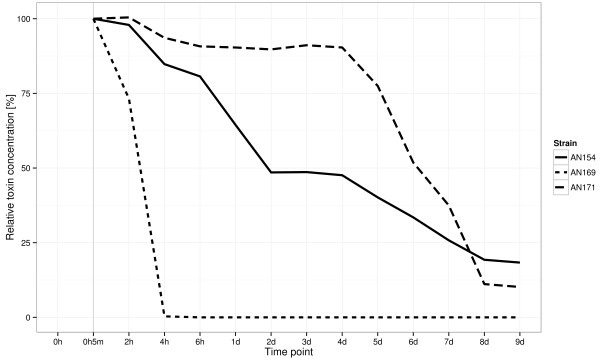
**Kinetic reduction of zearalenone during incubation experiments with isolates AN 154 (*C. rosea*), AN 169 (*C. catenulatum*) and AN 171 (*T. aggressivum*).** Experiments were carried out at 25°C, in liquid Czapek-Dox medium supplemented with yeast extract and zearalenone.

The fastest mycotoxin decomposition was observed in the isolate AN 169 (*C. catenulatum*), where after 24 hours the levels of ZEN were found to have declined below detectable levels (complete biotransformation ability). In the other two cases, the process progressed much slower. In case of isolate AN 154 (*C. rosea*), two days after incubation the concentration of ZEN decreased below 50% of initial concentration. In AN 171 culture (*T. aggressivum*) comparable level was achieved after six additional days. In both cases, after full eight days of incubation the concentration of ZEN in the medium dropped by approximately 80–90% (see Figure 2).

### Zearalenone lactonohydrolase gene expression in isolates of Clonostachys sp. and isolate of Trichoderma sp

**Figure 3:**
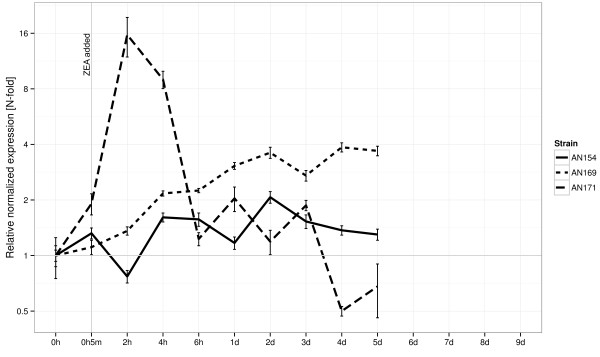
**Relative normalized expression (N-fold) of zearalenone lactonohydrolase transcripts during incubation experiments with isolates AN 154 (*C. rosea*), AN 169 (*C. catenulatum*) and AN 171 (*T. aggressivum*).** Experiments were carried out at 25°C, in liquid Czapek-Dox medium supplemented with yeast extract and zearalenone.

Expression of zearalenone lactonohydrolase gene was tested via quantitative RT-PCR (with β-tubulin as reference gene). The isolate AN 171 (*T. aggressivum*) isolate exhibited over 16-fold induced increase in *zhd101* expression 2 hours after zearalenone exposure (which corresponds with results of chemical analysis showing gradually expressed biotransformation ability potential). Conversely, the two other isolates AN 154 (*C. rosea*) and AN 169 (*C. catenulatum*) exhibited different expression patterns. The AN 169 isolate (the most effective detoxifier) accumulates higher transcript levels slowly but consistently over the period of days, while AN 154 most likely presents constitutive varying enzyme activity (as evidenced by low slope/plateaus in biotransformation ability process following fluctuations in transcript levels - see Figure 3).

### Phylogenetic analysis of gene sequences in multiple species

In unsupervised clustering of a/b-hydrolases from multiple genomes, newly sequenced homologs of zearalenone lactonohydrolase were found to form a stable cluster of sequences with previously published homologues from *B. ochroleuca*, as well as 2 additional proteins from *M. brunnea* and *A. montagnei*.

**Figure 4:**
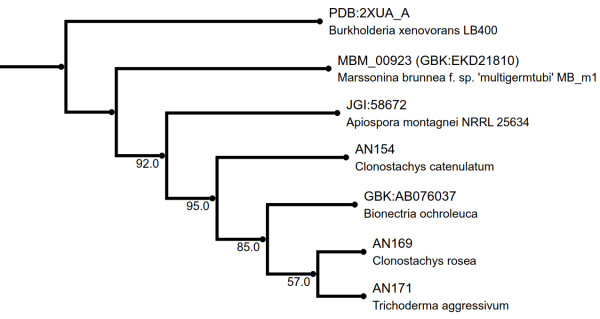
**Maximum likelihood phylogenetic tree of zearalenone lactonohydrolase homologs from divergent filamentous fungi.** Bootstrap support is indicated below bifurcations (1000 bootstrap iterations). Tree was based on 245 distinct patterns within a trimmed alignment of full length protein sequences (see: Methods section).

While phylogenetic reconstruction by maximum likelihood indicated strong support for a monophyletic clade formed by the cluster members (Figure 4), positioning of the resulting clade within a/b-hydrolase phylogeny was poorly supported and thus remains uncertain.

### Homology modelling and comparative structure analysis

**Figure 5:**
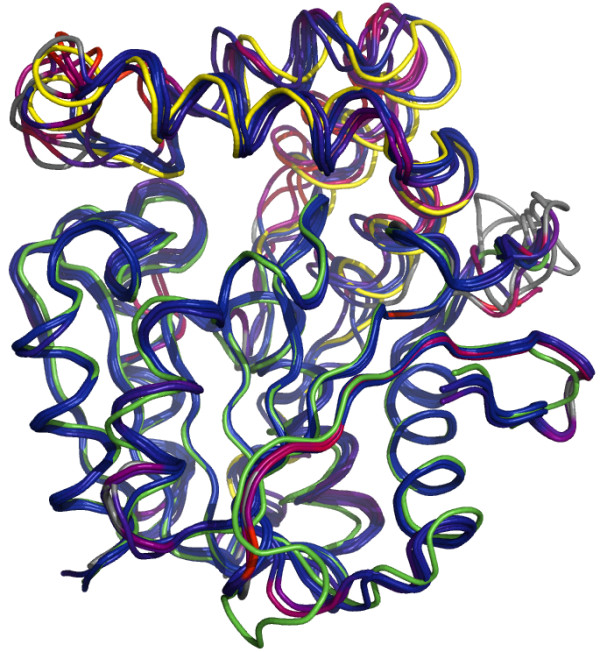
**Superposed structures of template 2XUA (3-oxoadipate lactonase; catalytic domain colored in green, cap domain colored in yellow) and homology models for zearalenone lactonohydrolase homologs from multiple species (see corresponding alignment on Figure**6**).** Coloring is based on RMSD between superposed Ca atoms (blue – best, red – worst; gray parts not included in superposition).

The created homology models uncovered similarities in the active site pocket, as detected by *fpocket*[15]. In all of the modelled structures, the active site pocket is strongly hydrophobic under normal conditions - likely the catalysis is enabled by allowing access to the active site (conformational changes involving cap domain) which allows the reaction to proceed by standard mechanism involving forming a transient oxyanion hole and subsequent cleavage of the lactone ring (Figure 5). While homology-based models are likely insufficient for elucidation of full sequence of events during substrate binding and catalysis (both the variable cap domain e.g. [16,17] and surrounding loops [18] are involved in controlling and fine-tuning substrate access), we were nevertheless able to ascertain the key functional residues involved.

**Figure 6:**
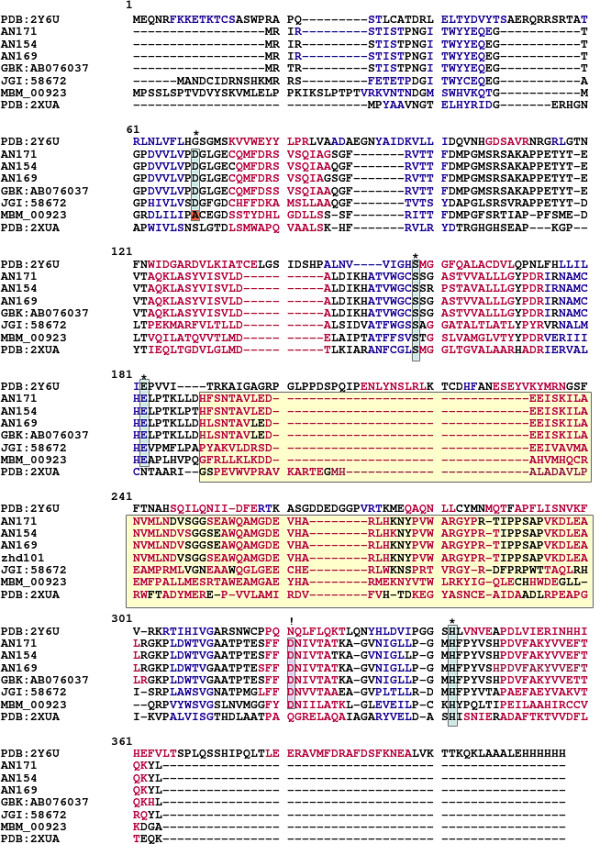
**Multiple alignment of protein sequences corresponding to: template structure 2XUA (3-oxoadipate lactonase), template structure 2Y6U (peroxisomal epoxide hydrolase Lpx1) and lactonase homologs from examined isolates (AN154, AN169, AN171), as well as reference sequences from *Bionectria ochroleuca *(GBK:AB076037), *Apiospora montagnei *(JGI:58672) and *Marsonnina brunnea *(MBM_00923 = GBK:EKD21810).** Proposed catalytic residues marked with ‘*’ (E128/D31, S102, H242). Residue D223 [11] marked with ‘!’. Secondary structure annotated based on PDB records (2XUA, 2Y6U) and RAPTORX 3-state SSE predictions (a-helix - red, b-sheet - blue). Predicted cap domain enclosed in yellow square.

**Figure 7:**
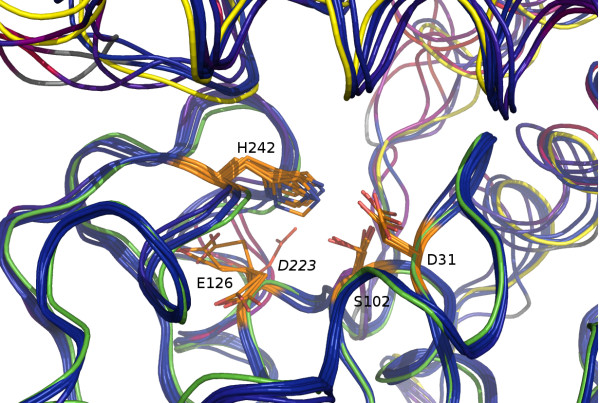
**Active site within superposed structures (see Figure**5**for description).** Modelled conformations of putative residues (S102, H242, E126/D31) involved in catalysis are coloured in orange, distal D223 (*B. ochroleuca*) proposed in earlier work [11] is shown in red.

Our identification of the catalytic triad conflicts with the initial proposition of Takahashi-Ando [11] that active site is formed by S102-H242-D223 (numeration by alignment in Figure 6). Typically, the nucleophilic attack of hydrolase enzyme is facilitated by interaction of histidine with acidic residue (third member of catalytic triad). This role, according to all our homology-based models cannot be fulfilled by D223 (residue located distantly to active site - Figure 7).

A typically, the third member of catalytic triad appears to be E126 residue, where the side chain is capable of interacting with distal nitrogen of catalytic histidine, provided conformational changes allow rotation of the glutamate side chain towards histidine (see Figure 5 for conformations in modelled structures). This residue is sequentially equivalent (see Figure 7) to catalytic glutamate residues demonstrated in human epoxide hydrolase (PDB:2Y6U, E153) and epoxide hydrolase from *Pseudomonas aeruginosa* (PDB:3KDA, E169).

Another possibility is residue D31 - however it appears to be nonconserved in *Marssonina* sequence (alanine substitution). Sequencing error cannot be completely ruled out in this case, as a single nucleotide change is sufficient for aspartate to alanine substitution in this context. Notably, D31 residue position in relation to the active site histidine favorises interactions with proximal imidazole nitrogen (mean distance of ca. 2.5 A^0^ across models) - suggesting possible conformational change (freeing the imidazole ring) during substrate binding.

## Discussion

Zearalenone is one of the most dangerous mycotoxins produced by fungi belonging to the *Fusarium* genus. Those species are usually severe pathogens of cereals and legumes, and may cause *Fusarium* head blight and *Fusarium* ear rot of corn. These toxins are contributing to significant economic losses in livestock production causing the disease known as estrogenic syndrome, which results in a sterility. Since 1988 [10] it is known that among the fungi of *Hypocreales* order, the mycoparasitic fungus *C. rosea* have the ability for zearalenone decomposition but so far no such properties has been described in any species of the *Trichoderma* genus.

Selected mycoparasitic *Trichoderma* and *Clonostachys* isolates were found to be able to reduce significantly both the production of zearalenone on medium Czapek-Dox broth with Yeast Extract [19] and to detoxify zearalenone. The three isolates (AN 154, AN 171 - especially AN 169) were clearly demonstrated as possible agents with verified biotransformation ability (*in vitro*). This finding includes the first demonstration of zearalenone lactonohydrolase activity present in a member of *Trichoderma* genus (AN 171 - *T. aggressivum*).

Both gene expression and the ability of isolate AN 171 (*T. aggressivum*) to reduce zearalenone levels were confirmed *in vitro* experiments. The HPLC experiments verified that all three isolates are able to decrease toxin concentration in a quick and effective manner [11]. The RT-PCR analyses further indicate that the expression of the zearalenone lactonohydrolase gene is subject to different modes of regulation in examined isolates. In particular, for the isolate AN 171, two hours after the toxin administration, a significant increase in the zearalenone lactonohydrolase expression is noted, suggesting that in *T. aggressivum* the presence of zearalenone in the medium directly activates expression of the gene.

Further study of sequence variation in lactonohydrolase genes is planned, with redesign of PCR markers based on sequenced regions and extension into non-coding regions of transcript (5′-UTR) [11] using RACE-PCR. Subsequent research will also encompass separation and identification of end products for detoxification process, as well as isolation of enzyme protein using Western blot.

Previous works have confirmed the existence and function of zearalenone - specific lactonase in *Clonostachys* sp. (old name of *Gliocladium* sp.) [9]. The enzyme is one of the reasons *Clonostachys* growth is not inhibited by zearalenone. We posit that presence of functioning homologues within *Trichoderma* can also contribute to their effective antagonistic activity [19], against zearalenone-producing *F. culmorum* and *F. graminearum* (and possibly other resorcyclic acid lactone producers).

Mechanistic features of catalytic site involved in zearalenone biotransformation ability are shown to be evolutionarily old, likely predating the split between *Leotiomycetes* and *Sordariomycetes* (barring horizontal transfer between fungal hosts). While it is unlikely that the exact function of distant homologs is the same, the affinity towards large hydrophobic epoxides and conservation of catalytic mechanism (as evidenced by active site superposition - Figure 7) are likely. Presence of several conserved arginines within the cap domain raises possibility of their involvement in substrate binding or orientation (coupled with conformational change), analogous to the mechanisms observed previously in dienelactone hydrolase [20] and 3-oxoadipate enol lactonase [16]. Elucidation of the full substrate orientation/catalysis scenario (including involvement of the glutamate and aspartate residues and their spatial conformations during the process) is planned through application of molecular dynamics experiments for modelling of the ligand binding process.

Notably, according to previously published work on *B. ochroleuca* enzyme [11] ZEN was rapidly replaced with conversion product. The mass of the molecular ion (M + 1) corresponding to this product was 293. In our analysis, we did not register the corresponding peak, either due to differences in protocol or because of another mechanism of zearalenone decomposition. An alternative mechanism of zearalenone biotransformation ability was recently demonstrated in basidiomycetous yeast *Trichosporon mycotoxinivorans*[11], which requires a modification of hypothetical ZOM-1 intermediate by an unspecified a/b-hydrolase. One possibility, testing of which is beyond the scope of current work, is that the one-step lactonohydrolase evolved as a neofunctionalisation (present within filamentous fungi of *Leotiomycetes*/*Sordariomycetes* orders) of the two-step detoxification mechanism retained by *T. mycotoxinivorans*. If so, the original mechanism can still exist in select extant lineages (within filamentous *Ascomycota*) in varying degrees (dependent on selection pressure towards one-step detoxification).

## Conclusions

Our research shows the first finding of a functional zearalenone lactonohydrolase in mycoparasitic *Trichoderma aggressivum* (an activity earlier characterised in the *Clonostachys rosea* strains). Based on the combined screening of over ninety isolates of *Trichoderma*/*Clonostachys* and *in silico* investigation of origins of the enzyme activity (through phylogeny reconstruction and homology modelling) we were able to provide supporting evidence for its evolutionary origins, as well as monophyly of functional lactonohydrolase homologs in both genera. The supporting evidence for presence and activity of functional enzyme homologs is based on chemical analyses, gene expression patterns, homology models showing conservation of key structural features and a marked reduction of zearalenone content in cultured samples (containing both medium and mycelium).

## Methods

### Fungal isolates

Fungal isolates originated from culture collections of the Institute of Plant Genetics (Polish Academy of Sciences, Poznan, Poland); Institute of Science of Food Production (Bari, Italy; ITEM), Institute of Food Technology (Poznan University of Life Sciences, Poznan, Poland), Department of Forest Pathology (Poznan University of Life Sciences, Poznan, Poland), Research Institute of Vegetable Crops, (Skierniewice, Poland) and Rothamsted International UK. The isolates were derived from soil, compost, wood, cultivated mushroom and cereal grain samples. All 98 isolates were identified using both morphological [21] and molecular methods (ITS 4-5 and *tef1* markers) (Additional file 1: Table S1).

### Isolation of pure cultures

Fungal isolates investigated in this study were collected from pieces of decaying wood, cultivated mushroom compost, samples of soil and cereal grain. The samples were plated on salt water nutrient agar (SNA) [22] and incubated at 20°C for 6 days. Putative *Trichoderma* and *Clonostachys* colonies were purified on potato dextrose agar (PDA, Oxoid). Pure culture were transferred to the tubes containing SNA medium and stored at -20°C for further study.

### Isolation of DNA

Mycelium used for DNA extraction was obtained by inoculating Czapek-Dox broth (Sigma-Aldrich) with Yeast Extract (Oxoid) and streptomycin sulphate (50 mg/L^-1^, AppliChem) and after incubation at 25°C for 21 days on a rotary shaker (120 rpm). Mycelium was collected on filter paper in a Büchner funnel, was held with sterile water, frozen at -20°C, and freeze - dried. Total DNA was extracted using the CTAB method [23]. The quality of DNA was estimated by NanoDrop 2000 UV-vis Spectrophotometer (Thermo Scientific, Wilmington, USA) and via Experion Automated Electrophoresis System (Bio-Rad, Hercules, CA).

### Primer design

In the case of *C. rosea* zearalenone lactonohydrolase, previous experiments performed by [9] suggested the use of degenerate starters for identification of homologous sequences. In our approach to direct sequencing of amplified fragments, degenerate primers gave only non-specific products. Because of that seven pairs of primers were designed on basis of available GenBank homologs (Table 1). The primers targeted evenly spread sites along the coding sequence (ca. 300 bp estimated product length; estimated melting temperature: 60°C). Primer pairs were designed in Primer 3 [24] and manually adjusted based on evaluation of melting parameters in OligoCalc [25].

**Table 1:** Sequences of the primers used for amplification and sequencing

**Primer name**	**Sequences (5′-3′)**	**Estimated product length**
LacDP26F	GAGCCAAGAGAGACCCACAG	
LacDP347R	TTATGTCCGAATGTCGTTGA	321
LacDP326F	GTTCAACGACATTCGGACAT	
LacDP712R	AACGTAGTGACCCTGAAGCC	386
LacDP693F	GGCTTCAGGGTCACTACGTT	
LacDP903R	GTATCCTGTCGGGGTAACCG	210
LacDP886F	GTTACCCCGACAGGATACGC	
LacDP1208R	GAAAGACTCGGTTGGTGTCG	322
LacDP1188F	GCGACACCAACCGAGTCTTT	
LacDP1400R	TACAATATCGCCTGCCCTCT	212
LacDP1380F	GAGAGGGCAGGCGATATTGT	
LacDP1695R	GGGAGCGAGTCAACAACCTA	315
LacDP1661F	AATCTCCGCCATGCTTAGG	
LacDP1990R	GGCTGGTCTCCCGTACAAT	329

### PCR amplification and sequencing

The PCR reaction was carried out in a 25 μl reaction mixture containing the following: 1 μl 50 ng/μl of DNA, 2.5 μl 10 × PCR buffer (50 mM KCl, 1.5 mM MgCl2, 10 mM Tris- HCl, pH8.8, 0.1% TritonX-100), 1.5 μ l00 mM dNTP (GH Healthcare), 0.2 μl 100 mM of each primer, 19.35 μl MQ H2O, 0.25 μl (2 U/μl) DyNAzyme TM II DNA Polymerase (Finnzymes). Amplifications were performed in C1000 thermocycler (BIO RAD, USA) under the following conditions: initial denaturation 5 min at 94°C, 35 cycles of 45 s at 94°C, 45 s at 56°C for all 7 pare primers, 1 min at 72°C, with the final extension of 10 min at 72°C. Amplification products were separated on 1.5% agarose gel (Invitrogen) in 1 × TBE buffer (0.178 M Tris-borate, 0.178 M boric acid, 0.004 M EDTA) and stained with ethidium bromide. The 10-μl PCR products were combined with 2 μl of loading buffer (0.25% bromophenolblue, 30% glycerol). A 100-bp DNA LadderPlus (Fermentas) was used as a size standard. PCR products were electrophoresed at 3 V cm^-1^ for about 2 h, visualized under UV light and photographed (Syngene UV visualizer). The 3 μl PCR products were purified with exonuclease I and shrimp alkaline phosphatase according to [26]. Sequencing reactions were prepare using the ABI Prism BigDye Terminator Cycle Sequencing ReadyReaction Kit in 5 μl volume (Applied Biosystems, Switzerland). DNA sequencing was performed on an ABI PRISM3100 GeneticAnalyzer (USA).

Sequences were edited and assembled using Chromas v.1.43 (Technelysium Pty Ltd). CLUSTAL W [27] and MUSCLE [28] were used to align the nucleotide sequences for comparison; the resulting alignments were inspected, merged and refined manually.

### RNA isolation and gene expression data analysis

Mycelium was collected from the Czapek-Dox medium. Each sample was weighted on laboratory scales (Sartorius). Total RNA was purified using RNeasy Plant Mini Kit (Qiagen, Hilden, Germany) according to the manufacturers’ protocol with the additional DNase digestion step. The quality of total RNA was estimated by Nanodrop (Thermo Scientific, Wilmington, DE) and via Bioanalyzer (Bio-Rad, Hercules CA).

The primer pairs specific to target gene were designed using zearalenone lactonohydrolase gene sequences obtained from *T. aggressivum*, *C. rosea*, *C. catenulatum* isolates (Table 2). Analogously to the DNA sequencing primers, these were designed with use of Primer 3 [24] and their properties were tested using OligoCalc [25].

**Table 2:** The sequences of the primers used for gene expression

**Primer**	**Sequences (5′-3′)**
LACDP723R	CAAACGTAGTGACCCTGAAGC
LACDP652F	CTCGGAGAATGCCAGATGTT
rtBtubTRICHOR2	AGCGAATCCGACCATGAAGA
rtBtubTRICHOF2	CACCGTCGTTGAGCCCTA

The RT-PCR reaction was conducted using SYBR® Green Quantitative RT-qPCR Kit (Sigma-Aldrich). The total reaction volume was 25 μl: 12.5 μl SYBR Green Taq Ready Mix, 1 μl RNA (< 35 ng), 0.5 μl each primer (10 μM), 0.125 μl reverse transcryptase and 5.125 μl nuclease free water. Gene expression profiles were determined through quantitative real-time PCR using a CFX96 Touch™ Real-Time PCR Detection System (Bio-Rad, Hercules, CA). The reaction was carried using the following protocol: initial denaturation 94°C for 2 min, followed by 40 cycles at 94°C for 15 s, 61°C for 1 min. The melting curve analysis (from 70°C to 95°C) confirmed primer pairs specificity. In the experiment we used three biological and two technical replicates together with a template-free negative control in each analysis of both target and control genes. As a control we used mycelium samples cultivated on medium without addition of zearalenone. Relative quantification of gene expression was done using the 2-_ΔΔ_Ct method (Bio-Rad, Hercules, CA). All data were normalized to β-tubulin as internal control (Real-Time PCR Application Guide, Bio-Rad, Hercules CA).

### Mycotoxin chemical analyses

#### Sample preparation

The fungal mycelium was grown in 50 ml Czapek-Dox broth (Sigma-Aldrich) with Yeast Extract (Oxoid) for 9 days at 25°C with rotary shaking at 100 rpm. The zearalenone (Sigma-Aldrich) stock was added after a week of incubation. The initial concentration of ZEA in the liquid cultures was 2 mg/ml. The samples (both mycelium and medium) were collected before and after addition of the toxin. During the first day, the samples were collected after one minute, two, four and six hours after toxin application. In the following days the samples were collected once a day at the same time. The collected material was used for both chemical analysis and expression profiling.

#### Chemicals and reagents

The zearalenone standard was supplied by Sigma-Aldrich-Aldrich (Steinheim, Germany). Acetonitrile and methanol (HPLC grade) were purchased from Sigma-Aldrich-Aldrich. Potassium chloride was purchased from Poch (Gliwice, Poland) and water (HPLC grade) was purified with a Millipore system (Billerica, MA, USA).

#### Zearalenone analysis

The samples (lysate containing both medium and mycelia) were filtered through glass microfibre filter (GF/B, Whatman). Zearalenone was analysed by the systems consisting of: Waters 2695 high-performance liquid chromatograph, Waters 2475 Multi λ Fluorescence Detector and Waters 2996 Photodiode Array Detector. Millenium software was used for data processing. The excitation wavelength and emission wavelength were set to 274 and 440 nm, respectively. The reversed-phase column C18 (150 mm × 3.9 mm, 4 μm particle, Waters) and acetonitrile-water-methanol (46:46:8, v/v/v) as the mobile phase at a flow rate 0.5 ml/min were used. Zearalenone quantification was performed by external calibration. The limit of zearalenone detection was 3 μg/kg.

The mass spectrometer (Esquire 3000, Bruker Daltonics, Bremen, Germany) was operating in the negative ions mode with an electrospray ion source (ESI) with the following settings: the source voltage 3860 V, nebulization with nitrogen at 30 psi, dry gas flow 9 L min^-1^, gas temperature 310°C, skimmer 1: -33 V, MS/MS fragmentation amplitude of 1 V ramping within the 40–400% range. Spectra were scanned in the mass range of m/z 50–700. The reversed-phase column was Alltima C18 (150 mm × 2 mm, 3 μm particle size) from Alltech. The column was kept at room temperature. Three biological and two technical replicates were used for each sample. The uninoculated medium with added toxin was used as a control.

### Database search and cluster analysis

The search for zearalenone lactonohydrolase homologues was conducted on internal, curated MetaSites database (Koczyk, unpublished). The dataset consisted of combined sequence data from translated GenBank release 192 (PLN and BCT divisions) [29], Ensembl/Fungi v 16 [30], UniProt/SwissProt [31], PDB [32] and sequences from select, published genomes from JGI/DOE MycoCosm [33]. Based on previous BLASTP searches for homologs of lactonohydrolase, a single homolog from unpublished genome of *A. montagnei* was included in the subsequent analysis.

The unsupervised cluster analysis was based on the subset of proteins detected by 2 iterations of NCBI PSI-BLAST [34], on the above-mentioned database clustered at 70% protein sequence identity with CD-HIT [35]. The zearalenone lactonohydrolase from *C. rosea* was employed as query. The unsupervised clustering of sequences (10728 total) was conducted in CLANS [36], using the neural-network based clustering option.

### Multiple alignment and phylogeny reconstruction

The preliminary alignment of a/b-hydrolases was prepared with MAFFT [37]. Conserved regions of the alignment were extracted with TrimAl based on 70% gap threshold setting [38]. Monophyly of the lactonhydrolase cluster within larger context of a/b-hydrolases was then assessed with FastTree2 [39] based on LG model (100 bootstraps) [40].

The multiple alignment of zearalenone lactonohydrolase cluster members was prepared using MAFFT-LINSI [37], and corrected manually in SeaView [41]. Conserved regions of the alignment were extracted with TrimAl using ‘*automated1*’ setting [38]. Maximum likelihood parameters were assessed with ProtTest v3 [42], according to Akaike and corrected Akaike information criterions. The phylogeny reconstruction for lactonhydrolase homologs was conducted in RAxML v 7.3 [43], using WAG model of evolution [44], with 1000 bootstrap iterations. Template sequence of the oxoadipate enol lactonase (PDB:2XUA) was employed as outgroup, in accordance with its ESTHER [45] classification in the epoxide hydrolase subgroup and its placement in homologs uncovered by HHpred [46].

Visualisation of the phylogenetic tree was prepared with ETE2 [47] and custom Python scripts.

### Homology modelling

Homology modelling was performed with RAPTOR-X webserver [48]. Choices of modelling templates were checked against HHpred [46] search results for candidate structures in pdb70 (with manual inspection of likely templates from epoxide hydrolase superfamily). HHpred was accessed via the MPI bioinformatics toolkit portal [49]. Visualisation and inspection of all models was conducted within PyMol [50]. All structure models are available in compressed form in Additional file 2. Multiple alignment of zearalenone lactonase homologs is available (in FASTA format) in Additional file 3.

## Competing interests

The authors declare that they have no competing interests.

## Authors’ contributions

DP and GK conceived the analysis, led the writing of this manuscript and production of figures and tables. DP and AD conducted the expression analyses and sequencing. KG performed the chemical analyses. GK performed the bioinformatic and phylogenetic analyses. LB and JC participated in drafting the manuscript and revising it critically. All authors read and approved the final manuscript.

## Supplementary Material

Additional file 1: Table S1 Examined isolates of *Trichoderma* and *Clonostachys.* Click here for file

Additional file 2 Structure models from homology modelling. Click here for file

Additional file 3 Multiple alignment of sequences in FASTA format. Click here for file

## References

[B1] Winssinger N Barluenga S Chemistry and biology of resorcylic acid lactones Chem Commun 2007 7 22 36 10.1039/b610344h 17279252

[B2] Zinedine A Soriano JM Moltó JC Mañes J Review on the toxicity, occurrence, metabolism, detoxification, regulations and intake of zearalenone: an oestrogenic mycotoxin Food Chem Toxicol 2007 45 1 18 10.1016/j.fct.2006.07.030 17045381

[B3] Ayed-Boussema I Ouanes Z Bacha H Abid S Toxicities induced in cultured cells exposed to zearalenone: apoptosis or mutagenesis? J Biochem Mol Toxicol 2007 21 136 144 10.1002/jbt.20171 17623888

[B4] Pfohl-Leszkowicz A Chekir-Ghedira L Bacha H Genotoxicity of zearalenone, an estrogenic mycotoxin: DNA adduct formation in female mouse tissues Carcinogenesis 1995 16 2315 2320 10.1093/carcin/16.10.2315 7586128

[B5] Chang K Kurtz HJ Mirocha CJ Effects of the mycotoxin zearalenone on swine reproduction Am J Vet Res 1979 40 1260 1267 525929

[B6] Gajecka M The effect of experimental low zearalenone intoxication on ovarian follicles in pre-pubertal bitches Pol J Vet Sci 2013 16 45 54 23691575

[B7] Tiemann U Dänicke S *In vivo* and *in vitro* effects of the mycotoxins zearalenone and deoxynivalenol on different non-reproductive and reproductive organs in female pigs: a review Food Addit Contam 2007 24 306 314 10.1080/02652030601053626 17364934

[B8] Karlovsky P Karlowsky P Secondary metabolites in soil ecology Soil Biology 2008 Berlin Heidelberg: Springer-Verlag 1 19

[B9] Utermark J Karlovsky P Role of zearalenone lactonase in protection of *Gliocladium roseum* from fungitoxic effects of the mycotoxin zearalenone Appl Environ Microbiol 2007 73 637 642 10.1128/AEM.01440-06 17114328 PMC1796959

[B10] el-Sharkawy S Abul-Hajj YJ Microbial cleavage of zearalenone. Xenobiotica Fate Foreign Compd Biol Syst 1988 18 365 371 10.3109/00498258809041672 2969647

[B11] Takahashi-Ando N Kimura M Kakeya H Osada H Yamaguchi I A novel lactonohydrolase responsible for the detoxification of zearalenone: enzyme purification and gene cloning Biochem J 2002 365 1 6 10.1042/BJ20020450 11978180 PMC1222652

[B12] Vekiru E Hametner C Mitterbauer R Rechthaler J Adam G Schatzmayr G Krska R Schuhmacher R Cleavage of zearalenone by *Trichosporon mycotoxinivorans* to a novel nonestrogenic metabolite Appl Environ Microbiol 2010 76 2353 2359 10.1128/AEM.01438-09 20118365 PMC2849244

[B13] Kriszt R Krifaton C Szoboszlay S Cserháti M Kriszt B Kukolya J Czéh A Fehér-Tóth S Török L Szőke Z Kovács KJ Barna T Ferenczi S A new zearalenone biodegradation strategy using non-pathogenic *Rhodococcus pyridinivorans* K408 strain Plos One 2012 7 e43608 10.1371/journal.pone.0043608 23049739 PMC3458049

[B14] Chen X Romaine CP Tan Q Schlagnhaufer B Ospina-Giraldo MD Royse DJ Huff DR PCR-based genotyping of epidemic and pre epidemic *Trichoderma* isolates associated with green mold of *Agaricus bisporus* Appl Environ Microbiol 1999 65 2674 2678 10347059 10.1128/aem.65.6.2674-2678.1999 PMC91394

[B15] Le Guilloux V Schmidtke P Tuffery P Fpocket: an open source platform for ligand pocket detection BMC Bioinformatics 2009 10 168 10.1186/1471-2105-10-168 19486540 PMC2700099

[B16] Bains J Kaufman L Farnell B Boulanger MJ A product analog bound form of 3-oxoadipate-enol-lactonase (PcaD) reveals a multifunctional role for the divergent cap domain J Mol Biol 2011 406 649 658 10.1016/j.jmb.2011.01.007 21237173

[B17] Nardini M Dijkstra BW Alpha/beta hydrolase fold enzymes: the family keeps growing Curr Opin Struct Biol 1999 9 732 737 10.1016/S0959-440X(99)00037-8 10607665

[B18] Li B Yang G Wu L Feng Y Role of the NC-loop in catalytic activity and stability in lipase from *Fervidobacterium changbaicum* Plos One 2012 7 e46881 10.1371/journal.pone.0046881 23056508 PMC3466181

[B19] Gromadzka K Chelkowski J Popiel D Kachlicki P Kostecki M Golinski P Solid substrate bioassay to evaluate the effect of *Trichoderma* and *Clonostachys* on the production of zearalenone by *Fusarium* species World Mycotoxin J 2009 2 45 52 10.3920/WMJ2008.x046

[B20] Cheah E Ashley GW Gary J Ollis D Catalysis by dienelactone hydrolase: a variation on the protease mechanism Proteins 1993 16 64 78 10.1002/prot.340160108 8497485

[B21] Błaszczyk L Popiel D Chełkowski J Koczyk G Samuels GJ Sobieralski K Siwulski M Species diversity of *Trichoderma* in Poland J Appl Genet 2011 52 233 243 10.1007/s13353-011-0039-z 21465156 PMC3088803

[B22] Nirenberg H Untersuchungen über die morphologische und biologische Differenzierung in der *Fusarium*-Sektion Liseola Mitteilungen Aus Biol Bundesanst Für Land- Forstwirtsch Berl-Dahl 1976 169 1 117

[B23] Doohan FM Parry DW Jenkinson P Nicholson P The use of species-specific PCR-based assays to analyse *Fusarium* ear blight of wheat Plant Pathol 1998 47 197 205 10.1046/j.1365-3059.1998.00218.x

[B24] Rozen S Skaletsky H Primer3 on the WWW for general users and for biologist programmers Methods Mol Biol 2000 132 365 386 10547847 10.1385/1-59259-192-2:365

[B25] Kibbe WA OligoCalc: an online oligonucleotide properties calculator Nucleic Acids Res 2007 35 W43 W46 10.1093/nar/gkm234 17452344 PMC1933198

[B26] Chełkowski J Golka L Stepień Ł Application of STS markers for leaf rust resistance genes in near-isogenic lines of spring wheat cv Thatcher. J Appl Genet 2003 44 323 338 12923307

[B27] Thompson JD Higgins DG Gibson TJ CLUSTAL W: improving the sensitivity of progressive multiple sequence alignment through sequence weighting, position-specific gap penalties and weight matrix choice Nucleic Acids Res 1994 22 4673 4680 10.1093/nar/22.22.4673 7984417 PMC308517

[B28] Edgar RC MUSCLE: a multiple sequence alignment method with reduced time and space complexity BMC Bioinformatics 2004 5 113 10.1186/1471-2105-5-113 15318951 PMC517706

[B29] Benson DA Cavanaugh M Clark K Karsch-Mizrachi I Lipman DJ Ostell J Sayers EW GenBank Nucleic Acids Res 2013 41 D36 42 10.1093/nar/gks1195 23193287 PMC3531190

[B30] Flicek P Ahmed I Amode MR Barrell D Beal K Brent S Carvalho-Silva D Clapham P Coates G Fairley S Fitzgerald S Gil L García-Girón C Gordon L Hourlier T Hunt S Juettemann T Kähäri AK Keenan S Komorowska M Kulesha E Longden I Maurel T McLaren WM Muffato M Nag R Overduin B Pignatelli M Pritchard B Pritchard E Ensembl 2013 Nucleic Acids Res 2013 41 D48 55 10.1093/nar/gks1236 23203987 PMC3531136

[B31] Consortium UP Update on activities at the Universal Protein Resource (UniProt) in 2013 Nucleic Acids Res 2013 41 D43 47 23161681 10.1093/nar/gks1068 PMC3531094

[B32] Rose PW Bi C Bluhm WF Christie CH Dimitropoulos D Dutta S Green RK Goodsell DS Prlic A Quesada M Quinn GB Ramos AG Westbrook JD Young J Zardecki C Berman HM Bourne PE The RCSB Protein Data Bank: new resources for research and education Nucleic Acids Res 2013 41 D475 482 10.1093/nar/gks1200 23193259 PMC3531086

[B33] Grigoriev IV Nordberg H Shabalov I Aerts A Cantor M Goodstein D Kuo A Minovitsky S Nikitin R Ohm RA Otillar R Poliakov A Ratnere I Riley R Smirnova T Rokhsar D Dubchak I The genome portal of the Department of Energy Joint Genome Institute Nucleic Acids Res 2012 40 D26 32 10.1093/nar/gkr947 22110030 PMC3245080

[B34] Schäffer AA Aravind L Madden TL Shavirin S Spouge JL Wolf YI Koonin EV Altschul SF Improving the accuracy of PSI-BLAST protein database searches with composition-based statistics and other refinements Nucleic Acids Res 2001 29 2994 3005 10.1093/nar/29.14.2994 11452024 PMC55814

[B35] Li W Godzik A Cd-hit: a fast program for clustering and comparing large sets of protein or nucleotide sequences Bioinforma 2006 22 1658 1659 10.1093/bioinformatics/btl158 16731699

[B36] Frickey T Lupas A CLANS: a Java application for visualizing protein families based on pairwise similarity Bioinforma 2004 20 3702 3704 10.1093/bioinformatics/bth444 15284097

[B37] Katoh K Toh H Parallelization of the MAFFT multiple sequence alignment program Bioinforma 1899–1900 2010 26 10.1093/bioinformatics/btq224 PMC2905546 20427515

[B38] Capella-Gutiérrez S Silla-Martínez JM Gabaldón T TrimAl: a tool for automated alignment trimming in large-scale phylogenetic analyses Bioinforma 1972–1973 2009 25 10.1093/bioinformatics/btp348 PMC2712344 19505945

[B39] Price MN Dehal PS Arkin AP FastTree 2-approximately maximum-likelihood trees for large alignments PLOS One 2010 5 e9490 10.1371/journal.pone.0009490 20224823 PMC2835736

[B40] Le SQ Gascuel O An improved general amino acid replacement matrix Mol Biol Evol 2008 25 1307 20 10.1093/molbev/msn067 18367465

[B41] Gouy M Guindon S Gascuel O SeaView version 4: a multiplatform graphical user interface for sequence alignment and phylogenetic tree building Mol Biol Evol 2010 27 221 224 10.1093/molbev/msp259 19854763

[B42] Darriba D Taboada GL Doallo R Posada D ProtTest 3: fast selection of best-fit models of protein evolution Bioinforma 2011 27 1164 1165 10.1093/bioinformatics/btr088 PMC5215816 21335321

[B43] Stamatakis A RAxML-VI-HPC: maximum likelihood-based phylogenetic analyses with thousands of taxa and mixed models Bioinforma 2006 22 2688 2690 10.1093/bioinformatics/btl446 16928733

[B44] Whelan S Goldman N A general empirical model of protein evolution derived from multiple protein families using a maximum-likelihood approach Mol Biol Evol 2001 18 691 699 10.1093/oxfordjournals.molbev.a003851 11319253

[B45] Lenfant N Hotelier T Velluet E Bourne Y Marchot P Chatonnet A ESTHER, the database of the α/β-hydrolase fold superfamily of proteins: tools to explore diversity of functions Nucleic Acids Res 2013 41 D423 429 10.1093/nar/gks1154 23193256 PMC3531081

[B46] Hildebrand A Remmert M Biegert A Söding J Fast and accurate automatic structure prediction with HHpred Proteins 2009 9 128 132 19626712 10.1002/prot.22499

[B47] Huerta-Cepas J Dopazo J Gabaldón T ETE: a python Environment for Tree Exploration BMC Bioinformatics 2010 11 24 10.1186/1471-2105-11-24 20070885 PMC2820433

[B48] Källberg M Wang H Wang S Peng J Wang Z Lu H Xu J Template-based protein structure modeling using the RaptorX web server Nat Protoc 2012 7 1511 1522 10.1038/nprot.2012.085 22814390 PMC4730388

[B49] Biegert A Mayer C Remmert M Söding J Lupas AN The MPI Bioinformatics Toolkit for protein sequence analysis Nucleic Acids Res 2006 34 335 339 10.1093/nar/gkl217 PMC1538786 16845021

[B50] Schrödinger L The PyMOL Molecular Graphics System, Version 1.3r1 2010

