# Supplementary material for: Zearalenone lactonohydrolase activity in Hypocreales and its evolutionary relationships within the epoxide hydrolase subset of a/b-hydrolases
Source: BMC Microbiol. 2014 Apr 3;14:82. doi: 10.1186/1471-2180-14-82 (PMC4021089; doi:10.1186/1471-2180-14-82)
Supplement: Additional file 1: Table S1 — Examined isolates of Trichoderma and Clonostachys. [file 1471-2180-14-82-S1.doc]

Table S1. Examined isolates of *Trichoderma* and *Clonostachys.*

| Culture code | Species | Sources/localization |
| --- | --- | --- |
| AN 4 | *T. harzianum* | oil tree, Italy |
| AN 13 | *T. atroviride* | forest soil, NPW^2^ |
| AN 14 | *T. atroviride* | forest soil, NPW |
| AN 19 | *T. atroviride* | forest soil, NPW |
| AN 21 | *T. hamatum* | forest soil, NPW |
| AN 22 | *T. gamsii* | forest soil, NPW |
| AN 35 | *T. atroviride* | maize kernels, Radzików |
| AN 46 | *T. citrinoviride* | soil, Poznań |
| AN 49 | *T. citrinoviride* | soil, Poznań |
| AN 55 | *T. polysporum* | soil, Poznań |
| AN 61 | *T. harzianum* | soil, Poznań |
| AN 68 | *T. virens* | compost, Puławy |
| AN 69 | *T. virens* | compost, Puławy |
| AN 70 | *T. virens* | compost, Puławy |
| AN 73 | *T. virens* | compost, Puławy |
| AN 74 | *T. virens* | compost, Puławy |
| AN 75 | *T. virens* | compost, Puławy |
| AN 89 | *T. citrinoviride* | garden soil, Poznań |
| AN 90 | *T. atroviride* | garden soil, Poznań |
| AN 91 | *T. harzianum* | compost, Poznań |
| AN 92 | *T. harzianum* | maize kernels, Radzików |
| AN 93 | *T. viridescens* | forest soil, Malta, Poznań |
| AN 94 | *T. harzianum* | forest soil, Malta Park, Poznań |
| AN 95 | *T. atroviride* | compost, Poznań |
| AN 96 | *T. atroviride* | compost, Poznań |
| AN 97 | *T. citrinoviride* | forest wood, Wieluń |
| AN 98 | *T. citrinoviride* | forest wood, Wieluń |
| AN 99 | *T. citrinoviride* | forest wood, Wieluń |
| AN 100 | *T. koningii* | forest wood, Wieluń |
| AN 101 | *T. harzianum* | forest wood, Wieluń |
| AN 102 | *T. citrinoviride* | forest wood, Wieluń |
| AN 104 | *T. koningii* | forest wood, Dziewicza Góra, Poznań |
| AN 105 | *T. koningii* | forest wood, Dziewicza Góra, Poznań |
| AN 106 | *T. koningii* | forest wood, Dziewicza Góra, Poznań |
| AN 107 | *T. koningii* | forest wood, Dziewicza Góra, Poznań |
| AN 108 | *T. harzianum* | forest wood, Dziewicza Góra, Poznań |
| AN 109 | *T. viride* | forest wood, Dziewicza Góra, Poznań |
| AN 110 | *T. harzianum* | forest wood, Dziewicza Góra, Poznań |
| AN 111 | *T. atroviride* | forest wood, Dziewicza Góra, Poznań |
| AN 113 | *T. koningii* | forest wood, Żurawiniec Park, Poznań |
| AN 114 | *T. koningii* | forest wood, Żurawiniec Park, Poznań |
| AN 115 | *T. koningii* | forest wood, Żurawiniec Park, Poznań |
| AN 116 | *T. koningii* | forest wood, Żurawiniec Park, Poznań |
| AN 117 | *T. koningii* | forest wood, Żurawiniec Park, Poznań |
| AN 118 | *T. hamatum* | forest wood, Rusałka Park, Poznań |
| AN 119 | *T. hamatum* | forest wood, Rusałka Park, Poznań |
| AN 120 | *T. hamatum* | forest wood, Rusałka Park, Poznań |
| AN 121 | *T. koningii* | forest wood, Rusałka Park, Poznań |
| AN 122 | *T. viridescens* | forest wood, Rusałka Park, Poznań |
| AN 124 | *T. koningii* | forest wood, Rusałka Park, Poznań |
| AN 125 | *T. koningii* | forest wood, Rusałka Park, Poznań |
| AN 126 | *T. koningii* | forest wood, Rusałka Park, Poznań |
| AN 127 | *T. koningii* | forest wood, Rusałka Park, Poznań |
| AN 128 | *T. koningii* | forest wood, Rusałka Park, Poznań |
| AN129 | *T. koningii* | forest wood, Rusałka Park, Poznań |
| AN 132 | *T. harzianum* | forest wood, Rusałka Park, Poznań |
| AN 133 | *T. harzianum* | forest wood, Jeziory, NPW |
| AN 134 | *T. harzianum* | forest wood, Jeziory, NPW |
| AN 135 | *T. harzianum* | forest wood, Jeziory, NPW |
| AN 136 | *T. harzianum* | forest wood, Jeziory, NPW |
| AN 137 | *T. harzianum* | forest wood, Jeziory, NPW |
| AN 138 | *T. harzianum* | forest wood, Jeziory, NPW |
| AN 141 | *T. viride* | forest wood, Jeziory, NPW |
| AN 142 | *T. viride* | forest wood, Jeziory, NPW |
| AN 143 | *T. koningiopsis* | forest wood, Jeziory, NPW |
| AN 144 | *T. koningii* | forest wood, Jeziory, NPW |
| AN 145 | *T. viridescens* | forest wood, Jeziory, NPW |
| AN 146 | *T. viridescens* | forest wood, Jeziory, NPW |
| AN 147 | *T. viridescens* | forest wood, Jeziory, NPW |
| AN 148 | *T. viridescens* | forest wood, Jeziory, NPW |
| AN 149 | *T. viridescens* | forest wood, Jeziory, NPW |
| AN 150 | *T. harzianum* | forest wood, Jeziory, NPW |
| AN 151 | *T. koningii* | forest wood, Jeziory, NPW |
| AN 152 | *T. atroviride* | triticale kernel, Choryń |
| AN 153 | *T. atroviride* | triticale kernel, Choryń |
| AN 155 | *T. hamatum* | rye rizosphera, Lublin |
| AN 158 | *T. koningiopsis* | triticale kernel, Lublin |
| AN 160 | *T. virens* | grass, Lublin |
| AN 171 | *T. aggressivum* | mushroom compost, Skierniewice, |
| AN 172 | T. aggressivum | mushroom compost, Skierniewice, |
| AN 173 | *T. viridescens* | oilseed rape, Poznań |
| AN 23 | *C. catenulatum* | forest wood, Zielonka |
| AN 25 | *C. rosea* | forest wood, Zielonka |
| AN 26 | *C. rosea* | forest wood, Zielonka |
| AN 32 | *C. catenulatum* | forest wood, Zielonka |
| AN 43 | *C. catenulatum* | forest wood, Zielonka |
| AN 78 | *C. rosea* | Soil, Harpenden, UK |
| AN 88 | *C. catenulatum* | Soli, Harpenden, UK |
| AN 154 | *C. rosea* | grass, Lublin |
| AN 161 | *C. rosea* | pease, Lublin |
| AN 162 | *G. fimbriatum* | soya bean, Lublin |
| AN 163 | *C. catenulatum* | soya bean, Lublin |
| AN 164 | *C. catenulatum* | beans, Lublin |
| AN 165 | *C. rosea* | pease, Lublin |
| AN 166 | *C. rosea* | pease, Lublin |
| AN 167 | *C. rosea* | beans, Lublin |
| AN 169 | *C. catenulatum* | beans, Lublin |
| AN 170 | *G. fimbriatum* | pease, Lublin |
